# Armoured Lepidopteran Caterpillars Preserved in Non-Fossil Resins and What They Tell Us about the Fossil Preservation of Caterpillars

**DOI:** 10.3390/insects15060380

**Published:** 2024-05-22

**Authors:** Joshua Gauweiler, André P. Amaral, Carolin Haug, Joachim T. Haug

**Affiliations:** 1 Cytology and Evolutionary Biology, Zoological Institute and Museum, University of Greifswald, Soldmannstraße 23, 17489 Greifswald, Germany; joshua.gauweiler@palaeo-evo-devo.info; 2 Biocenter, Ludwig-Maximilians-Universität München (LMU Munich), Großhaderner Str. 2, 82152 Planegg-Martinsried, Germany; andre.amaral@campus.lmu.de (A.P.A.); joachim.haug@palaeo-evo-devo.info (J.T.H.); 3 GeoBio-Center at LMU, Richard-Wagner-Str. 10, 80333 München, Germany

**Keywords:** Lepidoptera, copal, sub-fossil, defaunation resin, preservation

## Abstract

**Simple Summary:**

Some trees produce a plastic-like product called resin, which when fossilized is called amber. In this study we comment on the current terminological difficulties regarding the description of fossil, non-fossil and so called “sub-fossil” resins. We furthermore report two long-haired lepidopteran caterpillars in resin from Brazil and Madagascar. It is likely they represent larvae of Erebidae (tussock moths and others) which typically have long hairs and spines. Long-haired caterpillars are exceptionally rare in amber with only one similar specimen to date, as most other caterpillars in resin are either “naked” or have a protective case. These new specimens also increase the known size range of caterpillars preserved in resin to up to 12 mm. We also emphasize the importance of images when describing and publishing caterpillars in resin, to allow broader morphological studies using all available specimens.

**Abstract:**

Resin is a plastic-like product of trees. Older occurrences of such resin are referred to as amber and are considered fossil resin. Younger resins are termed copals. Even younger ones have been dubbed defaunation resins. Non-fossil resins remain in a terminological limbo, often referred to as “sub-fossils”. We report two lepidopteran caterpillars preserved in non-fossil resin: one from Madagascar, one from Brazil. Prominent hairs (=setae) and spines (=spine-like setae) of the specimens make it likely that they represent larvae of Erebidae (e.g., tussock moths and others). So far, most known caterpillars preserved in resins are either “naked” or bear protective cases; only few are armoured with spines or hairs. In particular, long-haired caterpillars such as the ones reported here are so far almost absent. Only one specimen with comparable setae has been reported from 15-million-year-old Dominican amber, but no significant details of this specimen are accessible. We briefly also review the record of caterpillars known from the Holocene, recognising that it is very sparse. The new specimens demonstrate that very hairy caterpillars can readily be preserved in resins in fine detail. Furthermore, the specimens increase the known size range of caterpillars preserved in resins, with one measuring more than 12 mm.

## 1. Introduction

Biodiversity has become a topic of interest for our society. In particular, the conservation of this increasingly endangered biodiversity has been recognised as an important task for humankind. To recognise changes in biodiversity, it is necessary to measure this diversity at different times and to compare them.

Short-term changes can be easily recognised by year-to-year surveys or by monitoring. Sources for longer-term comparisons can be older collections in museums, as many large-scale collections contain specimens collected in the field that have been conserved for many decades or even up a century. Even longer-term comparisons can involve fossils.

Furthermore, there is the conceptual difficulty in grasping the area between historical specimens and fossils. The term ”sub-fossil” has been used in many instances to address the remains of animals that are not (yet?) considered fossils. Cases from the literature include the following:

(1) Sub-fossil bones. For example, Holocene (the current geological epoch, starting ca. 11,700 years ago [1]) skulls of lemurs have been reported by Albrecht et al. [2] and dated to about 8245–1000 years before the present (see discussion in Albrecht et al. [2] (their p. 10)). These specimens differ from fossils in their mineralogy.
(2) Sub-fossil shells. For example, the shells of mussels described by Hjort & Funder [3]. These specimens, like the sub-fossil bones, are also from the Holocene, dating between 8500–5000 years before the present, and have different mineralogical properties than fossil shells.
(3) Sub-fossil plant remains. These can also be potentially differentiated from fossils by mineralogy. Yet, in some types of preservation other than petrification, such as charcoal formations [4], mineralogy does not play a major role. Hence, in some cases, age seems to be an important factor for characterising a sub-fossil. Examples of plant remains considered sub-fossils range from 6660–530 years before the present [4] (their p. 70), or even 7100–130 years before the present [5] (their Table 1, p. 298).
(4) Sub-fossil traces. For the interpretation of 9000–8000 years old mammalian tracks as sub-fossils, age was considered to be the main factor [6].
(5) Cuticle remains (specifically from representatives of Euarthropoda). Many organisms have cuticles that withstand relatively long time periods. Sub-fossil cuticle remains are known from different animals, including, for example, cladoceran crustaceans (water fleas) [7,8,9] and larvae of non-biting midges (Chironomidae) [10]. In many cases, the age of such remains, usually retrieved from lake sediments, is unknown or not reported. However, in a study involving the cuticle remains of larvae of non-biting midges, sub-fossils dating from 320 years before the present down to 10,000 years before the present were reported [11] (their table 2 p. 3347).

Despite the euarthropodan cuticle chemically degrading over time [12], some cuticle remains can persist for a long time. Preserved remains of small crustaceans have been reported from relatively recent times, such as the Neolithic period (12,000–6500 years ago, [13], [14] (their figure 15.2)), or from as late as the Mesozoic and the later Palaeozoic (e.g., [15,16]), possibly even the early Palaeozoic period [17]. The idea that fluorescence could be used to differentiate between extant and fossil remains (as suggested by Braun [15]) seems not to be universally applicable (e.g., scorpions show fluorescence today and in Silurian fossils [18]).

Hence, in many of the examples in which the term “sub-fossil” has been applied, they differ regarding their mineralogy, chemistry, and age, although most are restricted to the Holocene. Yet, there still seem to exist different views.

Schwartz et al. [19] (their p. 6124) used “recently extinct” as equivalent to sub-fossil. This imposes a difficulty, because some species preserved as sub-fossils (or fossils) might not be extinct (for example, see the discussion of beetles preserved in Miocene amber by Hörnschemeyer et al. [20]). This challenge, together with the different criteria used to identify a sub-fossil, shows that there is no well-formulated concept of what a sub-fossil is.

Another type of preservation often related to the term “sub-fossil” is copal. Copal is a type of resin known to bear inclusions, especially of representatives of various groups of Euarthropoda, and mainly of its ingroup Insecta. The assumed age of copal seems to differ greatly from author to author (e.g., [21] (his p. 440), [22] (their pp. 21–22), [1] (their figure 1, p. 2)); the transition point also varies greatly as to from which age a resin is considered a fossil resin, then commonly called amber (e.g., [1,23], [1] (their figure 1, p. 2)). Recently, Solórzano-Kraemer et al. [1] introduced another subdivision of non-fossil resins into an older copal and a younger defaunation resin.

We hereby report two pieces of non-fossil resins, one from Madagascar and one from Brazil, although it is unclear if they are copal or defaunation resins (see below for details). Each one includes a caterpillar-type larva of a lepidopteran. Caterpillars, in general, are comparably rare in the fossil record [24,25]. We discuss why these finds are of scientific interest, especially in taphonomy, and give a brief overview of the known Holocene record of lepidopteran caterpillars.

## 2. Materials and Methods

### 2.1. Materials

Two new specimens in non-fossil resins are at the centre of this study: one from Madagascar, one from Brazil. The specimen from Madagascar is preserved in a stalactite-like piece of non-fossil resin; this indicates that it might have been collected directly from the tree (see [22]). Therefore, it likely represents defaunation resin. For the specimen from Brazil, the resin origin is less clear; the shape of the copal does not give a hint as to whether it has been collected from the tree, from the ground, or elsewhere.

Both pieces do not contain obvious plant remains that could have been used for radiocarbon analyses (as in Solórzano-Kraemer et al. [1]). To address the uncertainty of whether the pieces are copal or defaunation resins, we will neutrally refer to the pieces as non-fossil resins to acknowledge the possibility that the pieces are not defaunation resin but copal (sensu Solórzano-Kraemer et al. [1]), although this seems unlikely. The pieces of non-fossil resin also contain other inclusions (e.g., numerous flies in the piece from Madagascar), but the largest inclusion in each piece is a caterpillar.

The piece from Madagascar was legally purchased from a trader (https://www.thefossildude.com/, accessed on 15 May 2024). A deposition in Madagascar was not (yet) possible. Therefore, it is now part of the Palaeo-Evo-Devo Research Group Collection of Arthropods, Ludwig-Maximilians-Universität München (LMU Munich), Germany, under repository number PED 0893.

The specimen from Brazil was legally purchased on the trading platform ebay.com from the trader meteoritestuff. It was temporarily part of the Palaeo-Evo-Devo Research Group Collection of Arthropods, Ludwig-Maximilians-Universität München (LMU Munich), Germany, under repository number PED 2014. It is now deposited at the Coleção Entomológica “Mítia Heusi Silveira”, at the Universidade Federal de Santa Catarina (UFSC), Brazil, under repository code CEMHS-D0054.

### 2.2. Methods

The specimens were documented on a VHX-6000 digital microscope on a black background and a white background. Different types of illumination were used (cross-polarised coaxial illumination, unpolarised ring illumination). Each image was additionally recorded under different exposure times (HDR; [26,27]). The best-contrasted ones were chosen for the figures in this study. All images represent composite images, i.e., to overcome limitations in the field of view and depth of field, adjacent image stacks were documented and processed following standard procedures (e.g., [28] and references therein). Furthermore, the specimens were documented in all accessible directions.

### 2.3. Basic Approach

As pointed out in the introduction, there are only few reports of lepidopteran caterpillars as fossils. We will list the first figured occurrence of a specimen in the literature and also detail in which articles or books it has been re-figured. Similar approaches have been used in other studies [24].

## 3. Results

**Figure 1:**
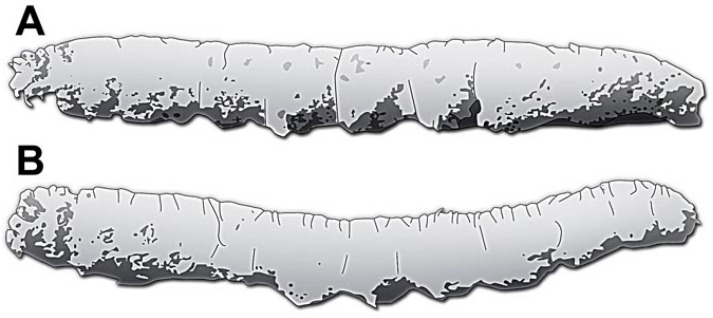
Drawings of the two mummified caterpillars reported by Keble [33], with the anterior end facing left, modified after Simonsen et al. [35], deposited in the Museum Victoria, Melbourne, Australia. (**A**) Specimen P16153, lateral view. (**B**) Specimen P16154, lateral view.

So far, there are only a few reports of lepidopteran caterpillars in the Holocene that can be considered sub-fossils:

(1) Rosenkjaer [29] reported possible larval cases of caterpillars from Denmark. The specimens have been discussed again by Henriksen [30]. Both references were not available in the original version but are cited indirectly from Sohn et al. [31]. Apparently, there were no figures associated with the reports. Sohn et al. [31] (their p. 35) stated that they “may represent the larval cases”. Hence, this stands a possible case, yet clearly an indirect one, as larval cases are often not easy to interpret. There is no additional information on the preservation given besides that the apparently isolated cases (likely not in resin) were found in unconsolidated sediments.
(2) Evers [32] reported lepidopteran specimens from resins that he referred to as copal. He depicted only winged adult forms. Yet, the author mentioned that he had seen three caterpillars preserved in these pieces of resin [32] (his p. 9).
(3) Keble [33] reported two caterpillars that had been replaced by fungus (his p. 49), representing “mummies” of the original morphology (Figure 1A,B). The specimens were depicted in Gill [34] (his p. 88 pl. III) and, in more detail, in Simonsen et al. [35] (p. 14, Figure 7). These “mummies” were not encased in resin.
(4) Lemdahl [36] reported isolated mandibles of caterpillars in sediments dated to between 9600–7900 years before the present [36] (his p. 307). No specimen was illustrated.

**Figure 2:**
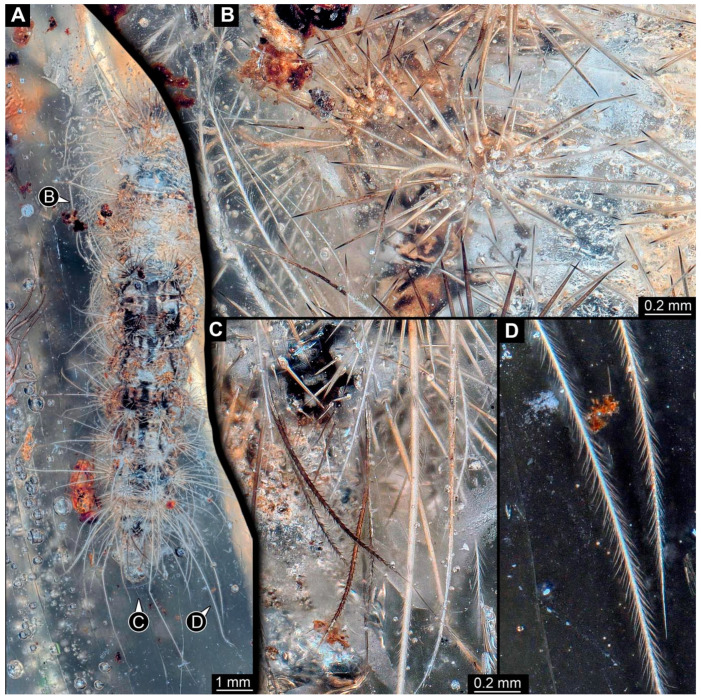
New specimen of a lepidopteran caterpillar in a non-fossil resin (PED 0893). (**A**) Specimen in dorsal view; the anterior part is not included in the amber. (**B**) Close-up of the larger humps on the abdomen with slightly fewer than 30 spine-like setae. (**C**) Close-up of the posterior body end; note the dark barbed setae. (**D**) Close-up of the long postero-lateral setae at the posterior body end; note the many small setules.

**Figure 3:**
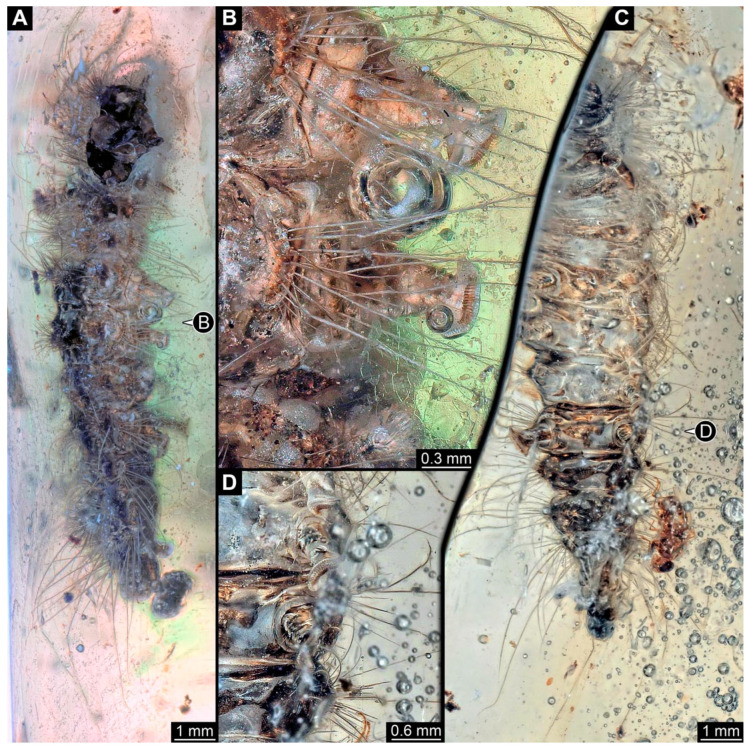
New specimen of a lepidopteran caterpillar in a non-fossil resin (PED 0893), continued. (**A**) Specimen in lateral view. (**B**) Close-up of the prolegs. (**C**) Specimen in ventral view; the anterior part is not included in the amber. (**D**) Close-up of the prolegs.

We additionally report two new specimens:

(5) The first new specimen from the non-fossil resin from Madagascar (PED 0893; Figure 2 and Figure 3) is preserved in quite clear resin. It is not fully preserved; the right anterior region is not inside the resin and therefore missing. The preserved part of the body is longer than 12 mm.

The specimen is elongated and tube-shaped, presumably with a head and trunk. The head seems largely missing. The trunk appears differentiated into the anterior thorax (three segments, only incompletely preserved) and posterior abdomen (nine segments + trunk end (possibly conjoined structure of segments 10 and 11)).

The visible thorax segments have four dorsal humps in a line. Each hump has about 20 spine-like setae (Figure 2A). There is a pair of smaller humps further posteriorly and medially. Each hump has about twelve spine-like setae (Figure 2A). The anterior eight abdomen segments have a slightly different armature: they have four larger and two smaller humps; the smaller humps are located antero-medially, the two larger humps antero-laterally, and the two larger humps postero-medially (Figure 2B). The larger humps have slightly fewer than 30 spine-like setae (Figure 2B). The larger humps have an additional single, very long softer seta (“hair”) with numerous setules (Figure 2D). The smaller humps have about ten spine-like setae. Abdomen segment 9 has a sub-similar armature. The postero-medial medial humps are without soft, setulae-bearing setae; instead, they have with slightly shorter, barbed setae (Figure 2C). The trunk end is elongated without prominent setae.

The habitus is less accessible in the lateral view. Each segment additionally bears a crescent-shaped elevation at the far lateral end. Each elevation has about ten short spine-like setae and about ten long softer setae with numerous setules (Figure 3A). Abdomen segments 3–6 each have a pair of appendages (“prolegs”). The appendages are organized into a proximally truncated and cone-shaped structure with few simple setae, distally widening to a trapezoid structure (“planta”) bearing about 20 hook-like spines (“crochets”) arranged into a single row (Figure 3B,D). The trunk end seems to bear a pair of appendages (“prolegs”); however, as the region is difficult to access, no details are visible. The habitus is hardly visible in the ventral view (Figure 3C).

**Figure 4:**
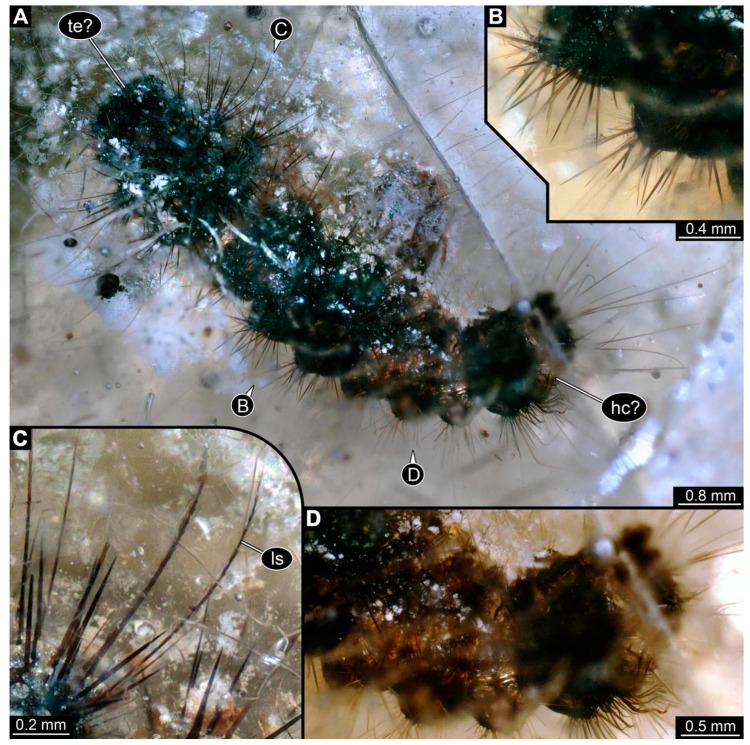
New specimen of a lepidopteran caterpillar in a non-fossil resin (CEMHS-D0054). (**A**) Overview of the specimen in the dorsal view. (**B**) Close-up of the lateral hump with short spines. (**C**) Close-up of the lateral hump with long setae, including additional small spines. (**D**) Close-up of what is likely to be the head capsule and anterior part of the body devoid of the dark green substance present elsewhere. Abbreviations: hc? = possible head capsule; ls = long barbed setae; te? = possible trunk end.

(6) The second specimen newly reported from a non-fossil resin from Brazil (CEMHS-D0054; Figure 4) is preserved in quite clear resin, but the piece has numerous cracks. The specimen is only visible in the dorsal view. The specimen seems fully preserved, but not many details are visible due to the brittle state of the resin (Figure 4A). The specimen is roughly 7.2 mm long. The head appears intact, but no details are visible. The trunk is differentiated into segments, but differentiation of the thorax and abdomen is difficult, since no ventral structures, especially legs, are available. The specimen has at least 13 major body units: the head, possibly 11 trunk segments, and the trunk end.

The anterior part of the specimen seems damaged (Figure 4D). Most of the dorsal side is covered by a dark green substance (it is unclear if this is only an optical interaction of the specimen and the resin or if this is a true substance). The anterior body region lacks this substance. All intact and accessible segments have distinct dorsal humps. The details are obscured, but each segment consistently has at least four large humps: two lateral, two dorsal.

The head bears around 20 setae with both multiple hair-like setae and short spine-like setae (Figure 4A). On the trunk segments, each lateral hump has at least 20 spine-like setae (Figure 4B). The supposed last three abdomen segments (not including the trunk end) bear, in addition to the spines, at least four long setae with small spine-like protruding setules (barbed setae; Figure 4C). Dorsal humps have an uncertain number of spines; the spines appear short, similar to the ones on the anterior lateral humps (Figure 4B). The trunk end has at least twelve short spines and at least three hair-like setae.

## 4. Discussion

### 4.1. Systematic Affinity of the Specimens

Specimen PED 0893 can be easily recognised as a lepidopteran larva (otherwise known as caterpillar) based on the presence of crochet-bearing prolegs on abdomen segments 3–6. With the numerous spines arising from dorsal humps, the specimen likely represents a type of heavily armoured caterpillar. It is not always easy to determine such armoured caterpillars (e.g., [37]).

The arrangements of spine-bearing humps, like those on the new specimen, are only seen in the larvae of two ingroups of Erebidae (Noctuoidea), namely Arctiinae (e.g., [38] (their pp. 70, 71, 73–84), [39] (their figures 1 and 3)) and Lymantriinae (e.g., [38] (their pp. 173–175), [40] (their figure 1D), [41] (his figure 1)). The observed barbed setae would support such a systematic affinity (see [41], his figure 3). Species of both groups (Arctiinae and Lymantriinae) are found in Madagascar and Brazil today [42] (his figure 13, p. 134), [43] (their table 6.1, p. 82), [44,45]; therefore, it is difficult to rule out either group based on the location they were found in. Since the specimen is also not visible in full detail, it is unclear whether it is a representative of either Arctiinae or Lymantriinae based on the morphology of the specimen. Hence, we tentatively interpret the specimen as a representative of Erebidae but make no statements on any more specific systematic affinity.

The second new specimen (CEMHS-D0054) could not be identified as easily. The limited access precludes the ability to identify any appendages and only allows an estimation of the general body segmentation and orientation of the specimen. Due to a similar arrangement of spine-bearing humps as in the first new specimen (PED 0893), we also tentatively interpret the specimen to be a representative of Erebidae, with no statements on any more specific systematic affinity for the same reasons as stated above.

### 4.2. The Lack of an Upper Boundary for the Fossil Record

The list of caterpillars from the Holocene is rather short. It could be much longer, depending on how one interprets the Holocene. This discrepancy is linked with the overall problem of the upper boundary of the fossil record, as well as with certain (partly politically driven) ideas on the younger Earth history (e.g., if an Anthropocene exists; see discussions in [46,47,48]).

In the literature, there appears to be no consensus on what a sub-fossil is (see Introduction). Additionally, the term “sub-recent” is not a better one, especially as “recent” has become a less-accepted term for a geological period (and should, in fact, not be used; [49] (their p. 1001)). In other words, there is no clear criterion as to when the term “sub-fossil” should be applied, which leaves us to a certain degree in a terminological limbo.

Especially for resins from Madagascar and Brazil (and other places; see Delclòs et al. [22]), this terminological uncertainty complicates things. Indeed, Delclòs et al. [22] recently demonstrated that these resins are very young. Using radiocarbon dating, they concluded that most of the Madagascar resins are likely between 80 and 300 years old and therefore not palaeontologically relevant. In fact, quite a number of specimens stored in alcohol in entomological museum collections may be older than some of the specimens preserved in natural resins. Thus, this type of resin is a non-fossil resin.

Additionally, there is a possibility that some animals preserved in copal are representatives of extant species [19]; in this case, some authors would not apply the term sub-fossil as they restrict the term to “recently extinct” species [50]. This contradiction, in fact, depends on the general problem of species in time. It has been argued that specimens preserved in Miocene Dominican amber might represent extant species [20]. Yet, there is actually no real criterion for evaluating such a statement. The major generally accepted species concepts lack any aspect of time, and even the few that include it do not provide an applicable way of how to deal with this problem. This does, in fact, not only apply to fossils or sub-fossils, but also to specimens stored in alcohol for decades (or more than a century). For more details on this discussion, refer to Haug & Haug [50].

### 4.3. Why These Specimens Are Still Informative

After Delclòs et al. [22] recognised how young Madagascar copal (and other copals) in fact is, they argued that it is therefore not of palaeo-biological value (we partly disagree on this point, as discussed further below), but that it is still valuable for other approaches. For example, it may prove important for shorter-term comparisons of biodiversity, especially concerning anthropogenic declines in biodiversity (further elaborated in Solórzano-Kraemer et al. [1]). Yet, Delclòs et al. [22] (their p. 24) also pointed out that for such a use, it will be important to know the exact provenance of the copal, but this is often not available, including for the specimens reported here. Still, these specimens provide some significant information.

Lepidopterans (moths and butterflies, although the latter is basically a derived form of the former) have generally been considered to be rare as fossils [31]. This rarity also extends to lepidopteran larvae, the caterpillars. That leaves the question of what the reasons are for this apparent rarity (especially in caterpillars). Clearly, caterpillars are rather soft, and likely rapidly decaying organisms, but amber and younger resins should clearly be capable of preserving caterpillars. Indeed, there are examples of caterpillars in amber. Yet, the numbers are still low. Only a handful of caterpillars are known from Cretaceous ambers [24,31,51,52], with slightly more specimens from Miocene ambers [25,31]. In Eocene Baltic amber, the numbers are higher; over 100 specimens seem to be known (e.g., [31,53,54,55,56,57,58,59,60,61,62,63,64]). Even so, this number is still low compared with the known number of specimens of other groups, such as the larvae of flies (Diptera; see discussion in Amaral et al. [65] and references therein) or the comparatively species-poor group of lacewings (Neuroptera; [25,66,67,68]). These scarce records give the impression that caterpillars are not easily trapped in resin to then become preserved in amber.

Curiously, many caterpillars preserved in amber seem to be “naked” caterpillars, i.e., having no or few indistinct spines and/or setae (see discussion in [24]). None of the caterpillars in Cretaceous ambers have long spines; in fact, only a single one is armoured at all, although with rather short, robust, and conical spines ([24,51] and references therein).

When looking at other holometabolan larvae in amber, we can see comparable situations, at least to a certain degree. Other caterpillar-type larvae with processes on their back, for example, those of scorpionflies (Mecoptera), are also rather small, although so far, the sample size is restricted to two specimens [52,69]. A larger sample size is available for lacewings (already used for a comparison in [25]), although the ecology is different from caterpillars as most lacewing larvae are predatory. Numerous fossil lacewing larvae possess prominent processes on their back [70,71,72,73,74,75,76,77]. Yet, the larger larvae [78,79], reaching into the centimetre range, lack such processes. For beetles, likewise, relatively many larvae have been found in amber, and some of these also bear processes [59], yet the really large beetle larvae in amber [80], measuring several centimetres, lack such processes. It seems, therefore, that large larvae with prominent processes (spines, hairs, etc.) are especially rare in resins, the two specimens reported here therefore being exceptionally important.

Among Eocene (Baltic amber) caterpillars, most seem to be of the naked type (e.g., [54] (their figure 335, p. 138, their figure 337, p. 139) and [54] (their p. 195, pl. 78a–c, d); the latter specimen was re-figured in [56] (their figure 13.48) and in [62] (their figure 7); [57] (his figure 145, p. 85), [60] (his figures 2567 and 7255, p. 340), re-figure from [53]; [61] (their Abb. 11.45, p. 77), re-figure from [60] (his figure 2567), [63] (their figure 1)), or they are case-building ones, which also lack spines ([53] (his figure 388, p. 139), [54] (their pl. 79 a–h, p. 197), [55] (their figure 3), [58] (their figures 1–12), [61] (their figures 1364, 2541, 2563, and 7532, p. 340, their figures 1374, 1384, 2503, 2524, and 7622, p. 341).

There is one exception in Baltic amber. A specimen reported by Poinar & Vega [64] (their figure 1A, B) has relatively long venomous setae, but it differs significantly from the specimen reported here in having shorter and fewer spines.

When looking into the Miocene, we also see several specimens of the naked type (including Mexican and Dominican amber; [81] (his figure F-216, p. 134), [56] (their figure 13.58), [82] (their figure 23), [83] (their figure 12), [84] (their figures 4–6, p. 150)) and case-bearing ones ([81] (his figures F-227 and F-228, p. 136), [56] (their figure 13.35)). In Dominican amber, more strongly armoured specimens have been found. A specimen reported by DeVries & Poinar [85] (their figure 1) (re-figured in [86] (their figure 107, colour plates 107, pp. 101–102) and also refigured in Poinar [87] (his figure 48)) has short, stubby, and balloon-like setae at the head–trunk transition and softer-appearing setae on the trunk. A more heavily armoured specimen in Dominican amber was reported by Poinar & Hammond [88] (their figures 1–3) (re-figured in [86] (their figure 53B, p. 58) and Poinar et al. [83] (their figure 13)). The spines are not very long, but they are prominent, carrying secondary spinules. Both specimens are less prominently armoured than the ones reported here.

A specimen with longer spines from Miocene Dominican amber was depicted in Poinar & Poinar [86] (their Figure 51, colour pl. 51, p. 55). Unlike the specimens we present, there seem to be fewer spine-like setae, and they appear not to be arranged in groups.

The only specimen more comparable to the ones here reported is from Miocene Dominican amber and was described in Wu [81] (his F-221, p. 135). It carries numerous long and strong setae and was also interpreted as a caterpillar of the group Erebidae (originally Lymantriidae, now ≈ Lymantriinae). Unfortunately, the image is rather small, which does allow for a closer inspection. Finally, one specimen preserved in presumably extant East African copal [32] was reported to have long setae and was interpreted as a possible larva of Lymantriidae (see [31] (their p. 81)).

Overall, the fossil record provides the impression that caterpillars are rarely preserved in amber, that naked caterpillars are more easily preserved than armoured specimens, and that heavily armoured specimens are virtually absent. There is no reason to believe that strongly armoured caterpillars with many spines and setae cannot be caught in resin. It is possible that these impressions are a reflection of their original abundance or simply a collection bias. The specimens reported here are therefore an interesting piece for actual-palaeontological consideration, which enable the present to be studied to better understand the past.

Additionally, the newly reported specimens provide us with information about the size range of caterpillars that can be preserved in resins. Notably, the preserved part of the body of the first specimen is more than 12 mm long. It now stands as the longest caterpillar preserved in resin reported so far (see recent review in [51]).

### 4.4. Show More Non-Fossil Resins, Please!

Non-fossil resins do, therefore, provide relatively important information concerning preservation in resins (see also Delclòs et al. [22]). However, to effectively use non-fossil resins in this way, it would obviously be necessary to have a much larger number of these types with inclusions, and for all of these inclusions to also be depicted when they are published. In the case of larvae, there is the general habit that immature ones are less likely to be published than adults, as there seems to be still a focus on new species descriptions based on adults (see discussion in Baranov et al. [89,90]). For non-fossil resins, the situation seems even worse. Delclòs et al. [22] mentioned more than 100 species described based on specimens preserved in copal (some of these would now likely be interpreted as defaunation resin). For larvae in copal, we have no numbers at hand; there is at least one lacewing larva in Colombian copal (defaunation resin?; [91] (his figure 206, p. 55)), but that seems to be an extreme exception. This tradition can also be seen in the case of Evers [32], who mentioned three caterpillars in his material at hand but did not illustrate a single one.

If non-fossil resins should indeed be used in diversity studies (as suggested in [22]), it would be necessary to have a much larger number of these to be accessible with images. Larvae, such as caterpillars, cannot be easily used in taxonomy-based approaches (for which a simple report of occurrence may often be sufficient), but they can be used in morphology-based studies ([68] and references therein). For such an approach, depicted specimens are necessary. Finally, as a last statement, we would like to add a suggestion to the community: please present more specimens, including those in copal and defaunation resins, as well as immature specimens.

## Data Availability

All data generated or analysed during this study are included in this published article.
